# Supplementary material for: Fractionalized conductivity and emergent self-duality near topological phase transitions
Source: Nat Commun. 2021 Sep 9;12:5347. doi: 10.1038/s41467-021-25707-z (PMC8429463; doi:10.1038/s41467-021-25707-z)
Supplement: Supplementary file 1 — Supplementary Information [file 41467_2021_25707_MOESM1_ESM.pdf]

Supplementary Information for: Fractionalized conductivity and emergent self-duality near topological phase transitions

Yan-Cheng Wang, Meng Cheng, William Witczak-Krempa, and Zi Yang Meng

In this supplementary Information, we present the discussion on the XY* quantum critical theory, in particular its operator content and the conductivity. We also present the two-step extrapolation of the quantum Monte Carlo data of conductivity from finite sizes and temperatures to the thermodynamic limit, and the fitting of the vison gaps from the imaginary time quantum Monte Carlo data, and their comparison with those obtained from stochastic analytic continuation.

Supplementary Note 1: XY* quantum critical theory.

We give a brief summary of the XY* quantum critical theory, focusing on the operator content in the continuum limit. The QCP is characterized by an emergent conformal symmetry, which means that it is described by a Conformal Field Theory (CFT) in 2 spatial and 1 time dimensions. Let us begin by describing the primary scaling operators in the regular XY (or O(2) Wilson-Fisher) CFT. They are labeled by their charge Q under the U(1) symmetry. First we discuss scalar (in the Lorentz sense) operators. Numerical conformal bootstrap and ϵ-expansion studies give the following values for the scaling dimensions of the first several primary operators [1, 2]:

$$\Delta_{Q=0} = 1.5117, \;\; \Delta_{Q=1} = 0.51926, \;\; \Delta_{Q=2} = 1.2357, \;\; \Delta_{Q=3} = 2.109, \;\; \Delta_{Q=4} > 3. \tag{1}$$

The last results two are from ϵ-expansion. These correspond to the operator O_Q with the lowest scaling dimension for the given charge.

We construct the XY* CFT by coupling the XY CFT to a $\mathbb{Z}_2$ gauge field, such that only even charges are gauge invariant. Namely, the gauge symmetry is $(-1)^Q$. In the new theory, all scalar primaries $\tilde{O}_Q$ are identified with O_{2Q} in the XY CFT, which are obviously closed under the Operator Product Expansion (OPE) algebra, and still form a consistent CFT. One must keep in mind that $\tilde{O}_Q$ still carries physical charge Q. For example, the charge-1 operator, which is related to $b^\dagger$ (S^+) in the BFG lattice model, has the scaling dimension $\tilde{\Delta}_1 = \Delta_2 = 1.2357$, corresponding to the anomalous dimension $\eta_1 = 2\tilde{\Delta}_1 - 1 = 1.4714$ (reasonably close to the QMC value is 1.53(4) [3, 4]). Interestingly, the minimal (positive) charge is now $Q = 1/2$, but the corresponding spinon operator is not gauge invariant. Physically, this tells us that physical states have an even number of spinons.

Now we consider operators with a Lorentz spin of 1 (vectors). Particularly important is the spin-1 current operator $\tilde{J}_\mu$. The integrated charge density $\tilde{J}_0$ inside a closed surface Σ, $\tilde{Q}(\Sigma)$, should satisfy

$$[\tilde{Q}(\Sigma), \tilde{O}_Q] = Q\tilde{O}_Q, \tag{2}$$

when the position of the operator $\tilde{O}_Q$ is inserted inside the volume enclosed by the surface. Since $\tilde{O}_Q = O_{2Q}$, we have

$$[Q(\Sigma), \tilde{O}_Q] = 2Q\tilde{O}_Q. \tag{3}$$

Consistency thus requires that we identify $\tilde{J}^\mu = \frac{1}{2}J^\mu$, so $\tilde{Q} = \frac{1}{2}Q$. This is expected since the fundamental scalar now carries charge-1/2.

In 2 spatial dimensions, the vacuum two-point correlation function of the current operator is given by (in imaginary time)

$$\langle J_\mu(x) J_\nu(0)\rangle = C_J \frac{\delta_{\mu\nu} - 2\hat{x}_\mu \hat{x}_\nu}{x^4}. \tag{4}$$

Here $\hat{x}_\mu = \frac{x_\mu}{|x|}$ and C_J is known as the current central charge. Using Kubo's formula, we can find that C_J is related to the universal groundstate conductivity by $\sigma(\infty) = \frac{\pi^2}{2} C_J$. For the XY CFT, numerical bootstrap yields $\sigma(\infty) \approx 0.3554$ [5].

Supplementary Note 2: Extrapolating the conductivity.

In Figs. 2 and 3 of the main text, we show the conductivity results (statistical errors are obtained from QMC simulations and standard data fitting) for inverse temperatures $\beta V = 300, 350, 390, 400, 450, 500, 520, 550, 600$ with system sizes $L = 12, 24, 36, 48, 60, 72, 96$ and extrapolate these data to the thermodynamic limit of $L \to \infty$ and then

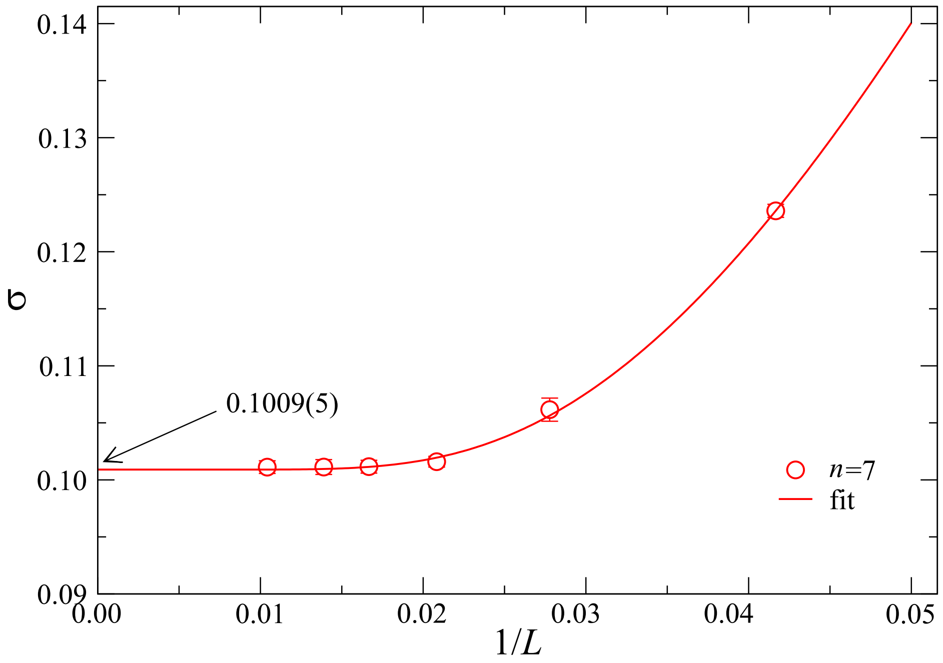

Supplementary Figure 1. **First extrapolation step for the conductivity.** The conductivity σ_n at $n = \omega_n/2\pi T = 7$ and boson filling $\langle n_i \rangle = 1/2$ as a function $1/L$ with $\beta V = 500$. After extrapolation to the thermodynamic limit with the fitting function $\sigma_n = \sigma_n(L \to \infty) + b \exp(-L/c)/\sqrt{L}$ (red line), one obtains $\sigma_n(L \to \infty, \beta V = 500) = 0.1009(5)$.

$\beta \to \infty$. The extrapolation is rather involved and here we explain the procedure in detail, taking the example of $\langle n_i \rangle = 1/2$, while the $\langle n_i \rangle = 1/3$ results are obtained with the same analysis (statistical errors are obtained using standard data fitting).

According to the previous analysis of the XY conductivity [6–8], the extrapolation to the thermodynamic shall be taken in two steps: first at a fixed inverse temperature β, one extrapolates the conductivity σ_n at every frequency ω_n to the system size $L \to \infty$, i.e. $\sigma_n(L \to \infty)$; then with the obtained $\sigma_n(L \to \infty)$, one performs the extrapolation of the inverse temperature $\beta \to \infty$, i.e. $\sigma_n(L \to \infty, \beta \to \infty)$. The obtained conductivity will be the one we use to extract the plateau value $\sigma(\infty)$ with the scaling function $\sigma_n(L \to \infty, \beta \to \infty) = \sigma(\infty) + b/n^{1.533} + c/n^3$, where 1.533 comes from $3 - 1/\nu$ with ν being the correlation length critical exponent. It takes the same value at both the XY* and XY QCPs: 0.67 [2].

The example of the first step extrapolation is shown in Supplementary Figure **1**, which is $\sigma(\omega_n)$ with $n = 7$ at $\beta V = 500$. Following the suggestion in Ref. [6, 7] we measure the conductivity at the configuration sector with no winding in the QMC world-line, in order to reduce the finite size effect. Then with $L = 12, 24, 36, 48, 60, 72, 96$ one sees that the $\sigma_{n=7}$ indeed converges to the fixed value with the fitting function $\sigma_n = \sigma_n(L \to \infty) + b \exp(-L/c)/\sqrt{L}$, where b and c are fitting parameters [7]. $\sigma_n(L \to \infty)$ of other frequencies are extrapolated in the same way at this β and we then proceed to the same $L \to \infty$ extrapolation for all the inverse temperatures $\beta V = 300, 350, 400, 450, 500, 600$.

The obtained $\sigma_n(L \to \infty)$ are shown in Fig. 2 of the main text. It is clearly seen that different curves are going towards the same limit as β increases. One interesting observation here is that at low frequencies, i.e. $n = 1, 2, 3, 4, 5$, the deviation between $\sigma_n(L \to \infty)$ at $\beta V = 300, 350$ with the lower temperature ones is big. Such nonmonotonous behavior is absent in the same analysis of the XY conductivity. This is due to the fact that at the XY* transition, the energy spectrum is more complicated than that of the XY transition, in particular with the presence of gapped vison excitations. In the kagome lattice Balents-Fisher-Girvin (BFG) model, previous works reveal that at the XY* transition where the spinon gap is closed, the vison excitations have a very small gap whose inverse is of the order of $\sim (V/t_c)^2 \approx 200$ [9]. Such a complication in the spectrum means that the extrapolation of the XY* data will be more difficult than that of the XY case and one cannot stay at relatively high temperature as in the case of XY transition but have to go significantly below the energy scale of the vison gap.

With such understanding of the complexity, we take the second step extrapolation of the inverse temperature β, mainly using the data of $\beta V \geq 300$. The extrapolation at two representative frequencies ω_n with $n = 10$ and 20 are shown in Supplementary Figure **2**. Here the extrapolation of β shall also follow a power-law, $\sigma_n(L \to \infty) = \sigma_n(L \to \infty, \beta \to \infty) + b/\beta^\omega$ [6, 7], where $\sigma_n(L \to \infty, \beta \to \infty)$ is the final extrapolated conductivity at this frequency, and

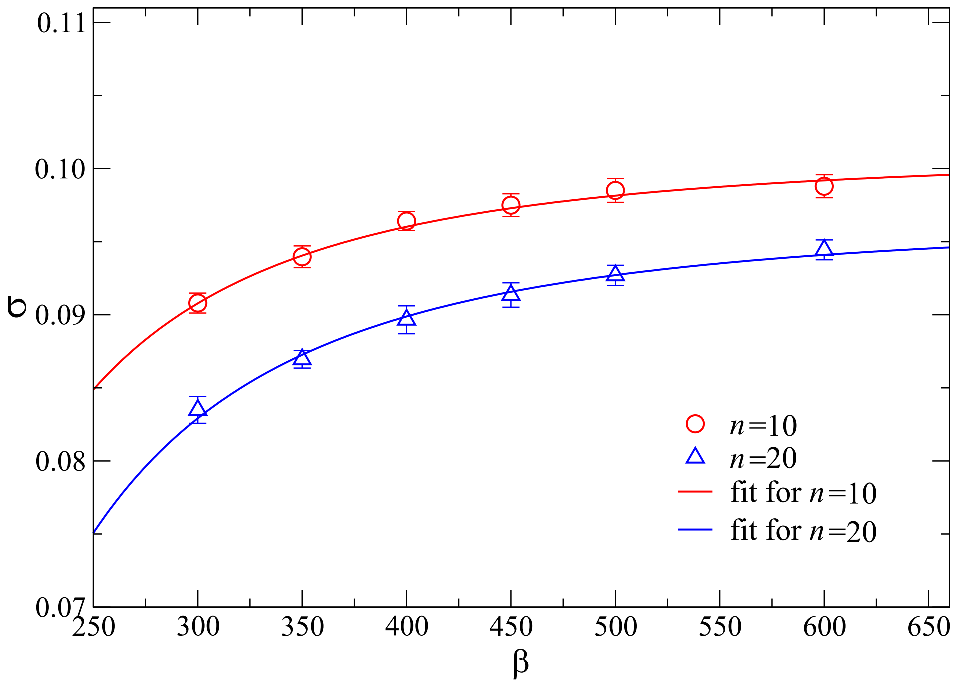

Supplementary Figure 2. **Second extrapolation step for the conductivity.** Two examples of β extrapolation of $\sigma_n(L \to \infty, \beta \to \infty)$ at $n = 10$ and $n = 20$. The fitting function is $\sigma_n(L \to \infty) = \sigma_n(L \to \infty, \beta \to \infty) + b/\beta^{\omega}$ [6, 7]. We find the effective correction exponent $\omega = 2.5$ gives the best fit for all the frequencies, when considering the data point $\beta \geq 300$ to avoid the vison gap. The extrapolated values are $\sigma_{n=10}(L \to \infty, \beta \to \infty) = 0.101(1)$ and $\sigma_{n=20}(L \to \infty, \beta \to \infty) = 0.097(1)$.

b is a fitting parameter and the ω is an effective exponent taking care of the corrections to the scaling, such scaling analyses have been successfully applied in the previous literatures on the conductivity extrapolation of superfluid-Mott insulator transitons [6–8]. We find that a larger $\omega = 2.5$ is a good choice such that the fitting curve can go through all the data points at $\beta V = 300, 350, 400, 450, 500$ and 600 for all the frequencies. Note that our choice of ω is larger than the effective value of ~ 0.9 used in the XY case, this is again the signature of the complexity of the critical spectra at the XY* transition. With such power-law form, one obtains $\sigma_{n=10}(L \to \infty, \beta \to \infty) = 0.101(1)$ and $\sigma_{n=20}(L \to \infty, \beta \to \infty) = 0.097(1)$, as shown in Supplementary Figure **2**. We further apply the same procedure for all the frequencies and the obtained $\sigma_n(L \to \infty, \beta \to \infty)$ are shown in Fig. 2 of the main text.

Finally, we fit the data in Fig. 2 of the main text with the scaling function $\sigma_n(L \to \infty, \beta \to \infty) = \sigma(\infty) + b/n^{1.533} + c/n^3$, with the (2+1)D O(2) correlation length exponent $\nu = 0.67$ plugged in. By choosing the frequency range of $n \in [1, 20]$, we can obtain the plateau value $\sigma(\infty) = 0.098(9)$ as discussed in the main text, whose value is within error bars 1/4 of the XY $\sigma(\infty)$. The coefficients b and c are found to depend very sensitively on the range of frequency used in the fit, but the overall sign structure, i.e. $b > 0$ and $c < 0$, is the same as those of the XY case. The ambiguity in b and c is likely due to the complex energy spectra at the XY* transition, especially the presence of the small vison gap. To resolve this issue, one would need to simulate the system at a much lower temperature and with larger system sizes, which are clearly beyond the scope of the current study and actually pose a challenging problem in terms of performing the next-generation QMC simulation schemes. We will leave such tasks to future works with more efficient algorithms and powerful supercomputing platforms.

Supplementary Note 3: Obtaining the vison gap.

The vison-pair spectra shown in Fig. 4 of the main text are obtained using the QMC-SAC scheme, and we have revealed the gapped spectra at the $\langle n_i \rangle = 1/2$ and $\langle n_i \rangle = 1/3$ XY* transition points. In fact, the presence of the vison gap can be directly read from the raw QMC data of the imaginary time decay of the dynamic spin correlation functions $S(\mathbf{q}, \tau)$, which is the spatial Fourier transform of $\langle S_i^z(\tau) S_j^z(0) \rangle$.

As shown in Supplementary Figure **3**, we present such $S(\mathbf{q}, \tau)$ at $\langle n_i \rangle = 1/3$ XY* transition with $L = 18$ and $\beta = 600$ for few representative momenta (with statistical errors obtained from QMC simulations). The exponential decay can be clearly seen and we fit the vison-pair gap with $S(\mathbf{q}, \tau) \sim a \exp(-\Delta(\mathbf{q})\tau)$ for the datas of $\tau \geq 40$ and the gap at different momenta can therefore be fitted.

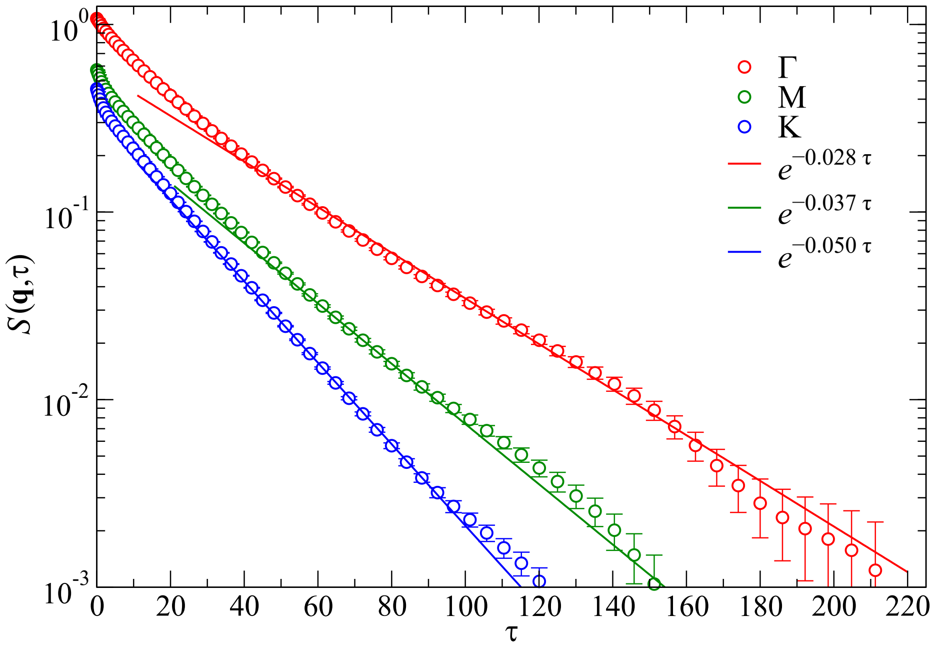

Supplementary Figure 3. **Obtaining the vison gap in imaginary time.** Structure factor $S(\mathbf{q},\tau)$ as function of τ at $\mathbf{q} = \Gamma, M$ and $q = K$ for the XY* transition at $\langle n_i \rangle = 1/3$ case with $L = 18$ and $\beta = 600$. The fitting function is $ae^{-\Delta(\mathbf{q})\tau}$ (data with $\tau \geq 40$ is used in the fit) and the obtained gap are shown in the figure.

Since the vison spectra are continua, we find the obtained value of the fitted gap in Supplementary Figure **3** are consistent with those seen in the analytic continued spectrum in Fig. 4(b) of the main text, in that, the fitted values correspond to the position between the lower edge to the brightest spectral weights in the continua. For example, the $\Delta(\Gamma) \sim 0.028$ and $\Delta(K) \sim 0.05$. This is another verification of the robustness of the QMC-SAC scheme employed here.

[1] Poland, D., Rychkov, S. and Vichi, A. The conformal bootstrap: Theory, numerical techniques, and applications. *Rev. Mod. Phys.* **91**, 015002 (2019).

[2] Chester, S. M. *et al.* Carving out OPE space and precise O(2) model critical exponents. *J. Hig. Ener. Phys.* **2020**, 2142 (2020).

[3] Isakov, S. V., Melko, R. G. and Hastings, M. B. Universal Signatures of Fractionalized Quantum Critical Points. *Science* **335**, 193 (2012).

[4] Wang, Y.-C., Fang, C., Cheng, M., Qi, Y. and Meng, Z. Y. Topological spin liquid with symmetry-protected edge states. *arXiv e-prints* p. arXiv:1701.01552 (2017).

[5] Reehorst, M., Trevisani, E. and Vichi, A. Mixed scalar-current bootstrap in three dimensions. *J. Hig. Ener. Phys.* **2020**, 156 (2020).

[6] Witczak-Krempa, W., Sørensen, E. S. and Sachdev, S. The dynamics of quantum criticality revealed by quantum Monte Carlo and holography. *Nat. Phys.* **10**, 361 (2014).

[7] Chen, K., Liu, L., Deng, Y., Pollet, L. and Prokof'ev, N. Universal conductivity in a two-dimensional superfluid-to-insulator quantum critical system. *Phys. Rev. Lett.* **112**, 030402 (2014).

[8] Katz, E., Sachdev, S., Sørensen, E. S. and Witczak-Krempa, W. Conformal field theories at nonzero temperature: Operator product expansions, Monte Carlo, and holography. *Phys. Rev. B* **90**, 245109 (2014).

[9] Sun, G.-Y., Wang, Y C., Fang, C., Cheng, M., Qi, Y. and Meng, Z. Y. Dynamical signature of symmetry fractionalization in frustrated magnets. *et al.*, *Phys. Rev. Lett.* **121**, 077201 (2018).
